# Supplementary material for: Brain age identification from diffusion MRI synergistically predicts neurodegenerative disease
Source: Imaging Neurosci (Camb). 2025 Apr 24;3:imag_a_00552. doi: 10.1162/imag_a_00552 (PMC12320004; doi:10.1162/imag_a_00552)
Supplement: Supplementary Material [file imag_a_00552-supp.pdf]

Brain age identification from diffusion MRI synergistically predicts neurodegenerative disease: Supplementary Materials

Supplementary figures & tables

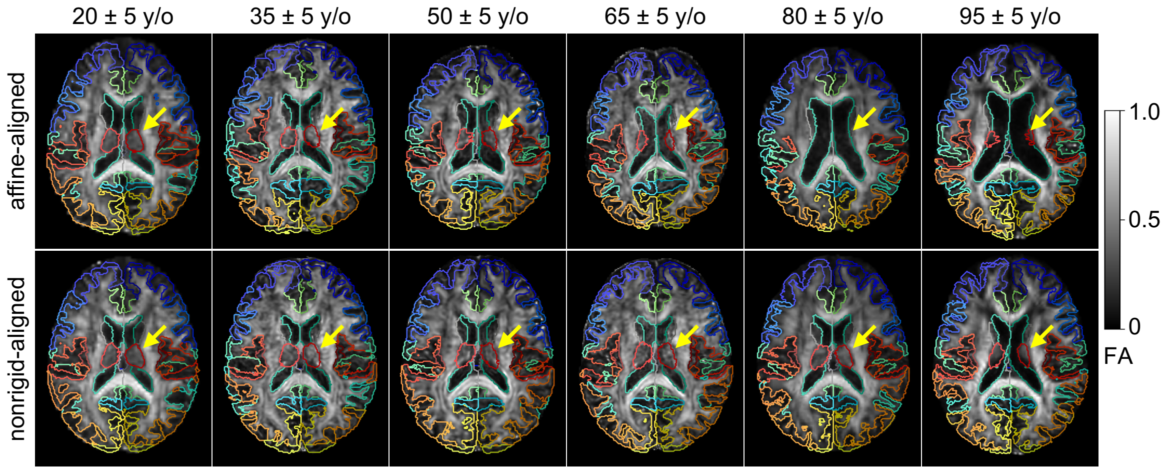

Figure S1. The macrostructural variations are present in the affine-aligned fractional anisotropy (FA) images, while mitigated in the nonrigid-aligned images. Contours of regions are provided to assist in the visual inspection of brain region shapes. Yellow arrows indicate the thalamus, which appears to shrink with age in the first row (affine-aligned) but remains consistent in shape and size in the second row (nonrigid-aligned).

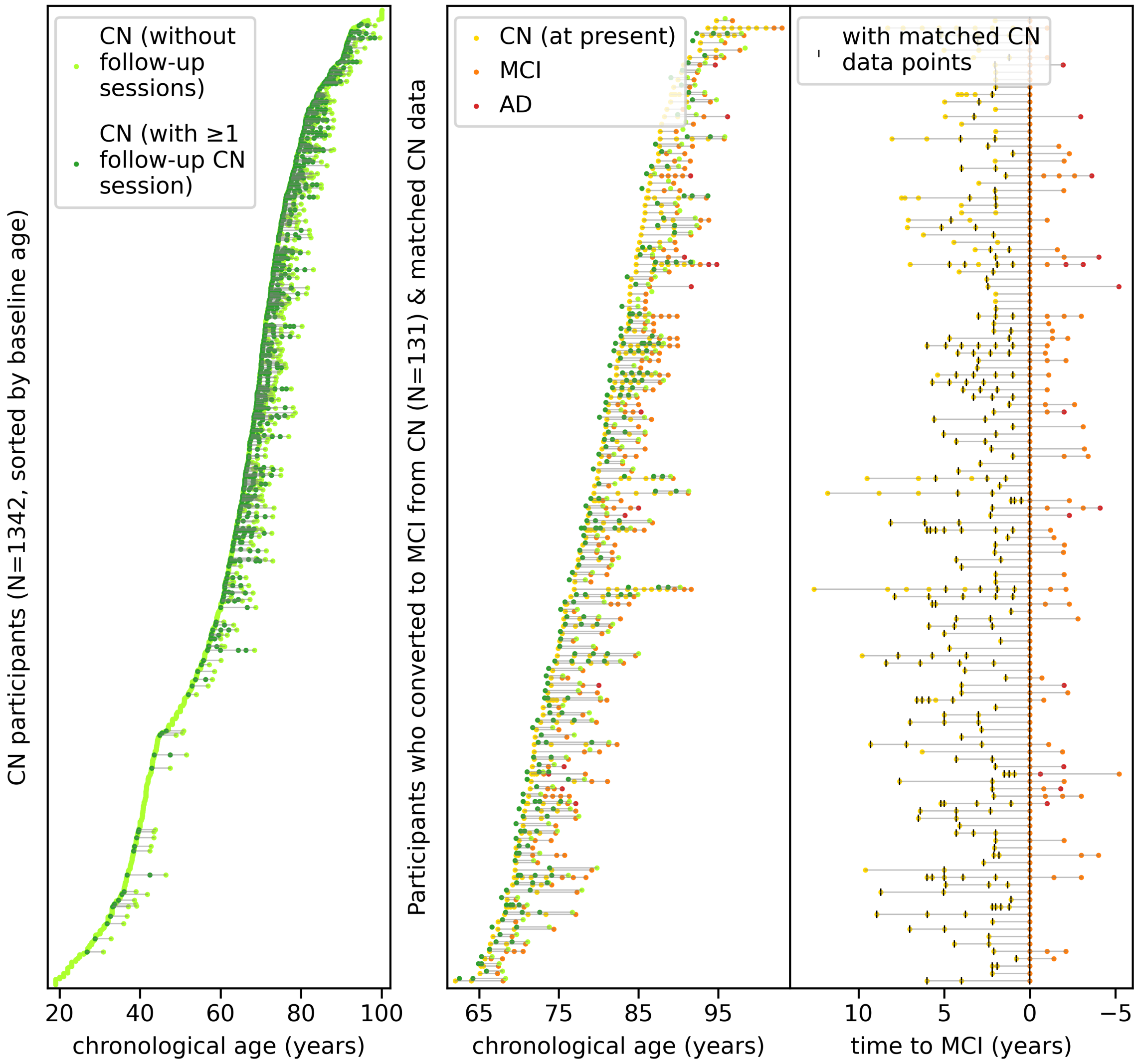

Figure S2. We match CN data points for participants who converted from CN to MCI based on sex, age, and time to event (i.e., time to first MCI diagnosis for MCI participants and time to last CN session for CN participants).

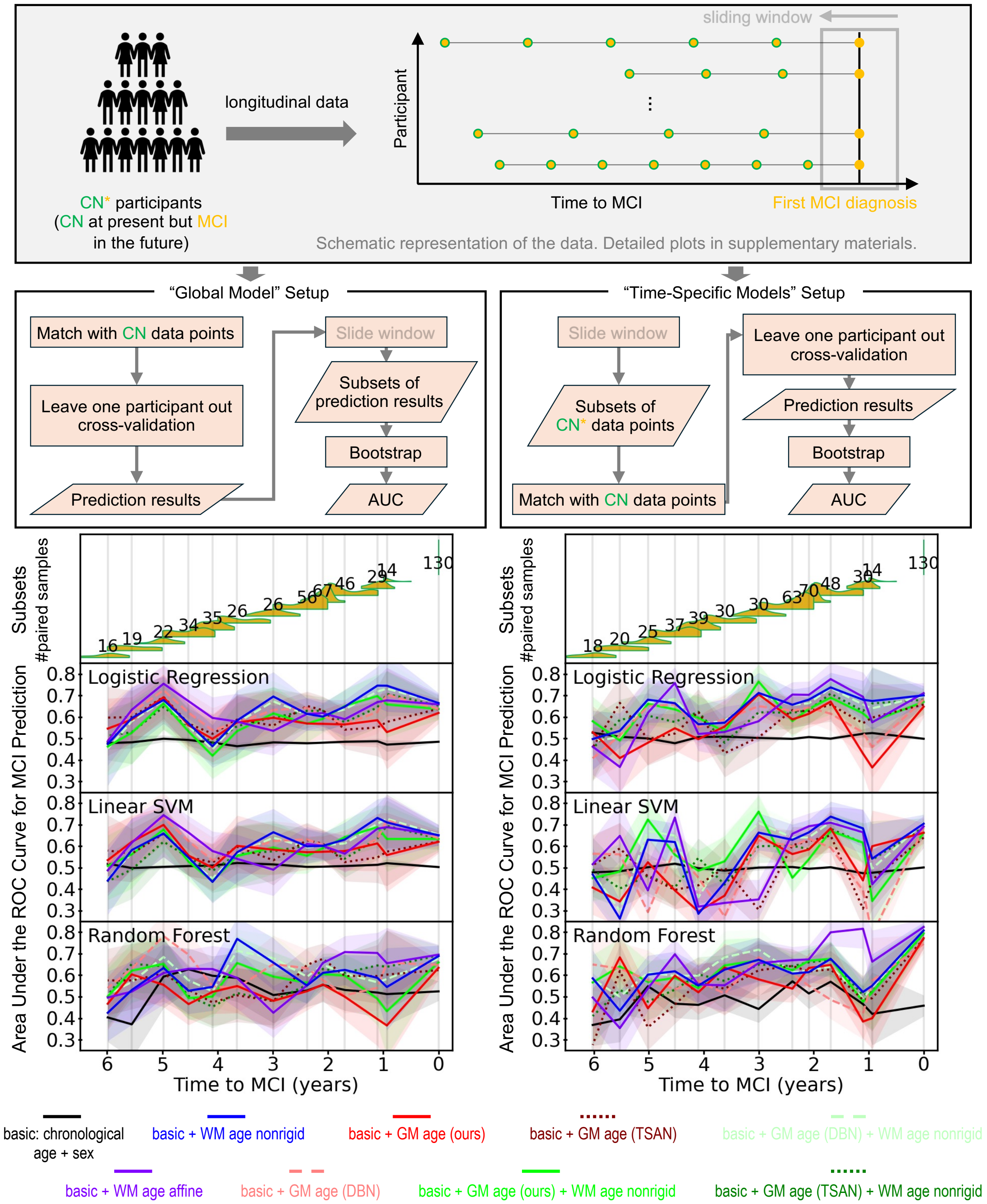

Figure S3. The longitudinal data from CN* participants are used for MCI prediction from n years pre-diagnosis in two experimental setups. In the "Global Model" setup, WM age nonrigid shows an advantage from 0 to approximately 3.5 years before MCI. In the "Time-Specific Models" setup, WM age nonrigid shows an advantage up to approximately 4–5 years before MCI. However, these advantages are not statistically significant, as indicated by the overlapping confidence intervals.

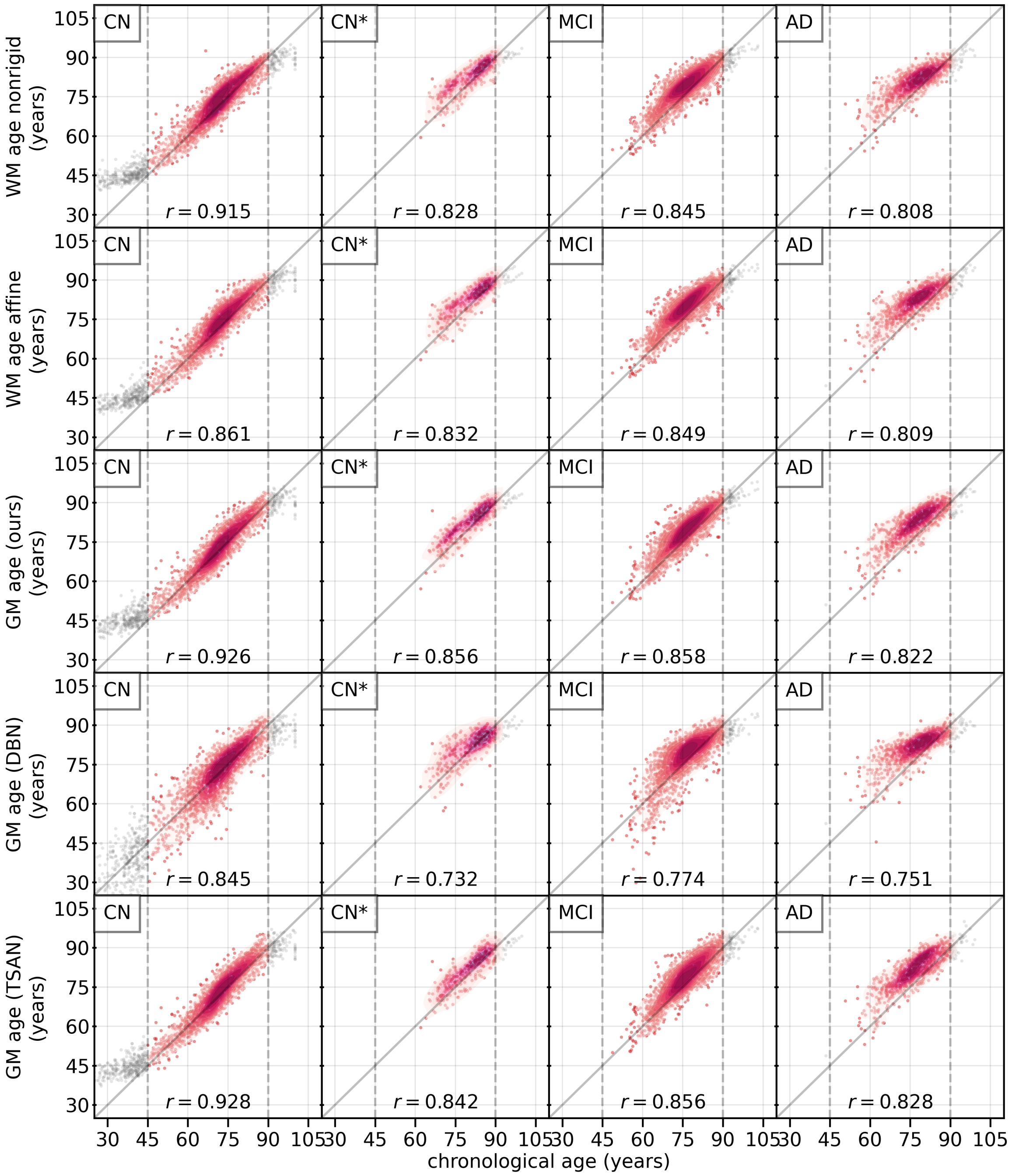

Figure S4. Estimated brain age vs. chronological age for participants that are (from left to right column) CN (cognitively normal), CN* (cognitively normal at present but transitioning to mild cognitive impairment), MCI (mild cognitive impairment), and AD (Alzheimer's disease). The Pearson correlation coefficients are calculated on data points within the age range of 45 to 90 years.

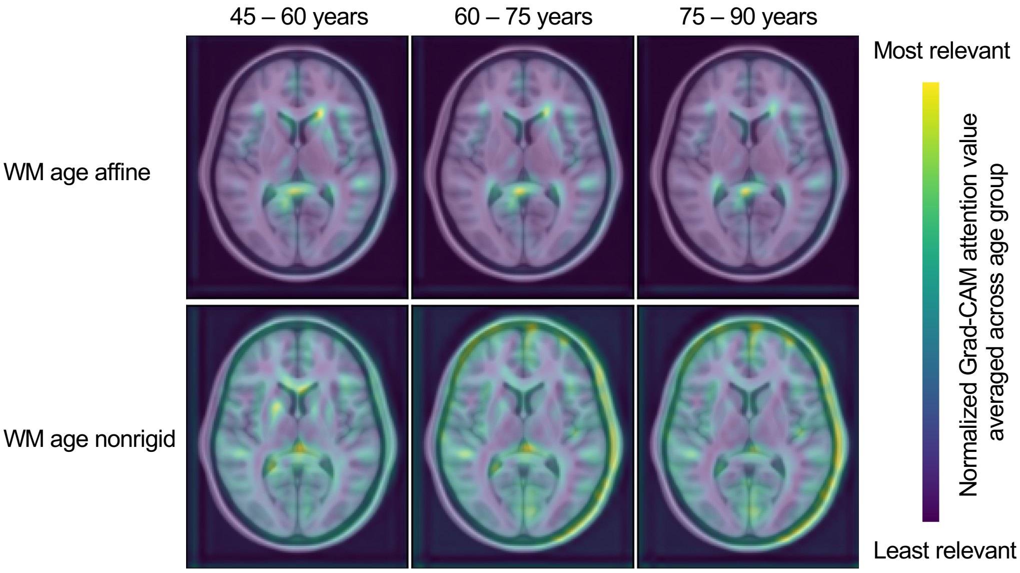

Figure S5. We compute Grad-CAM attention for WM age affine and WM age nonrigid, respectively. The attention values are normalized for each image, averaged across images from the same age group, and overlaid on the MNI152 template image. WM age nonrigid is driven by more regions beyond the ventricles, in contrast to WM age affine.

Table S1. Life table for the survival analysis.

Interval (years)	n CN at Beginning of Interval	n MCI During Interval	n Censored
0-2	421	19	29
2-4	373	49	127
4-6	197	34	86
6-8	77	17	26
8-10	34	10	21
10-12	3	1	1
12-14	1	1	0

Table S2. Classification of CN vs. AD, CN vs. MCI, and CN vs. CN* using chronological age, sex, and brain age-related features. To facilitate easier visual inspection, we use blue for WM age nonrigid, purple for WM age affine, red for GM ages, and green for combinations of GM ages and WM age nonrigid. The same color scheme is followed in other figures.

	CN vs. AD (N=458 matched pairs)					
	Logistic Regression		Linear SVM		Random Forest	
Features	Accuracy	AUC	Accuracy	AUC	Accuracy	AUC
basic: chronological age + sex	0.50 (0.50, 0.50)	0.50 (0.50, 0.50)	0.50 (0.50, 0.50)	0.50 (0.50, 0.50)	0.56 (0.55, 0.58)	0.59 (0.57, 0.60)
+ WM age nonrigid	0.65 (0.62, 0.67)	0.72 (0.70, 0.74)	0.65 (0.63, 0.67)	0.72 (0.70, 0.74)	0.69 (0.67, 0.72)	0.77 (0.74, 0.79)
+ WM age affine	0.67 (0.64, 0.69)	0.74 (0.72, 0.77)	0.67 (0.65, 0.70)	0.74 (0.72, 0.76)	0.69 (0.67, 0.71)	0.77 (0.74, 0.79)
+ GM age (ours)	0.69 (0.67, 0.71)	0.76 (0.74, 0.79)	0.69 (0.66, 0.71)	0.76 (0.73, 0.78)	0.70 (0.68, 0.73)	0.78 (0.76, 0.80)
+ GM age (DBN)	0.70 (0.68, 0.73)	0.78 (0.76, 0.80)	0.70 (0.68, 0.73)	0.78 (0.76, 0.80)	0.69 (0.67, 0.71)	0.77 (0.75, 0.79)
+ GM age (TSAN)	0.68 (0.66, 0.71)	0.76 (0.74, 0.78)	0.68 (0.66, 0.71)	0.75 (0.73, 0.78)	0.68 (0.66, 0.70)	0.76 (0.74, 0.78)
+ GM age (ours) + WM age nonrigid	0.70 (0.68, 0.72)	0.76 (0.74, 0.78)	0.70 (0.67, 0.72)	0.75 (0.73, 0.78)	0.73 (0.71, 0.75)	0.79 (0.77, 0.82)
+ GM age (DBN) + WM age nonrigid	**0.70 (0.68, 0.73)**	**0.79 (0.77, 0.81)**	**0.71 (0.69, 0.74)**	**0.79 (0.76, 0.81)**	**0.74 (0.72, 0.76)**	**0.81 (0.79, 0.83)**
+ GM age (TSAN) + WM age nonrigid	0.68 (0.66, 0.70)	0.76 (0.74, 0.78)	0.70 (0.68, 0.72)	0.76 (0.73, 0.78)	0.72 (0.69, 0.74)	0.79 (0.77, 0.81)

	CN vs. MCI (N=694 matched pairs)					
	Logistic Regression		Linear SVM		Random Forest	
Features	Accuracy	AUC	Accuracy	AUC	Accuracy	AUC
basic: chronological age + sex	0.50 (0.50, 0.50)	0.50 (0.50, 0.50)	0.50 (0.50, 0.50)	0.50 (0.50, 0.50)	0.57 (0.56, 0.59)	0.59 (0.58, 0.61)
+ WM age nonrigid	0.61 (0.59, 0.63)	0.65 (0.62, 0.67)	0.60 (0.59, 0.62)	0.65 (0.62, 0.67)	0.64 (0.62, 0.66)	0.70 (0.68, 0.72)
+ WM age affine	0.59 (0.57, 0.61)	0.63 (0.61, 0.65)	0.60 (0.58, 0.62)	0.63 (0.61, 0.65)	0.66 (0.64, 0.68)	0.69 (0.67, 0.71)
+ GM age (ours)	0.62 (0.60, 0.63)	0.65 (0.63, 0.67)	0.60 (0.58, 0.62)	0.64 (0.61, 0.66)	0.67 (0.65, 0.69)	0.72 (0.70, 0.74)
+ GM age (DBN)	0.59 (0.57, 0.61)	0.62 (0.59, 0.64)	0.58 (0.56, 0.60)	0.61 (0.59, 0.63)	0.59 (0.57, 0.61)	0.63 (0.61, 0.65)
+ GM age (TSAN)	0.59 (0.57, 0.61)	0.63 (0.61, 0.65)	0.57 (0.56, 0.60)	0.62 (0.60, 0.64)	0.63 (0.61, 0.65)	0.68 (0.65, 0.70)
+ GM age (ours) + WM age nonrigid	**0.62 (0.60, 0.64)**	**0.66 (0.64, 0.68)**	**0.63 (0.61, 0.65)**	**0.66 (0.64, 0.68)**	**0.67 (0.65, 0.69)**	**0.73 (0.71, 0.75)**
+ GM age (DBN) + WM age nonrigid	0.61 (0.59, 0.63)	0.65 (0.63, 0.67)	0.61 (0.59, 0.62)	0.65 (0.63, 0.67)	0.66 (0.64, 0.68)	0.72 (0.70, 0.74)
+ GM age (TSAN) + WM age nonrigid	0.60 (0.58, 0.62)	0.65 (0.63, 0.67)	0.60 (0.58, 0.62)	0.64 (0.62, 0.67)	0.66 (0.65, 0.68)	0.71 (0.69, 0.73)

	CN vs. CN* (N=118 matched pairs)					
	Logistic Regression		Linear SVM		Random Forest	
Features	Accuracy	AUC	Accuracy	AUC	Accuracy	AUC
basic: chronological age + sex	0.50 (0.50, 0.51)	0.50 (0.50, 0.50)	0.50 (0.50, 0.50)	0.50 (0.50, 0.50)	0.53 (0.50, 0.57)	0.56 (0.52, 0.60)
+ WM age nonrigid	0.60 (0.56, 0.65)	0.64 (0.59, 0.69)	**0.62 (0.57, 0.67)**	0.64 (0.59, 0.69)	0.61 (0.56, 0.66)	0.63 (0.58, 0.68)
+ WM age affine	0.61 (0.56, 0.66)	**0.64 (0.59, 0.70)**	0.59 (0.54, 0.64)	0.63 (0.58, 0.69)	0.55 (0.50, 0.59)	0.60 (0.55, 0.66)
+ GM age (ours)	0.60 (0.56, 0.65)	0.64 (0.59, 0.69)	0.62 (0.57, 0.67)	0.63 (0.57, 0.69)	0.60 (0.55, 0.64)	0.63 (0.57, 0.68)
+ GM age (DBN)	0.56 (0.51, 0.61)	0.62 (0.56, 0.67)	0.57 (0.52, 0.62)	0.61 (0.56, 0.66)	0.57 (0.53, 0.62)	0.59 (0.54, 0.65)
+ GM age (TSAN)	0.57 (0.53, 0.62)	0.62 (0.57, 0.68)	0.58 (0.54, 0.63)	0.61 (0.56, 0.67)	0.57 (0.52, 0.62)	0.58 (0.52, 0.63)
+ GM age (ours) + WM age nonrigid	0.60 (0.55, 0.65)	0.64 (0.59, 0.69)	0.61 (0.56, 0.65)	0.64 (0.59, 0.69)	0.61 (0.56, 0.65)	0.65 (0.60, 0.70)
+ GM age (DBN) + WM age nonrigid	**0.62 (0.57, 0.66)**	0.64 (0.59, 0.69)	0.60 (0.56, 0.65)	**0.65 (0.59, 0.70)**	**0.61 (0.57, 0.66)**	**0.66 (0.61, 0.71)**
+ GM age (TSAN) + WM age nonrigid	0.60 (0.55, 0.64)	0.64 (0.59, 0.69)	0.58 (0.53, 0.63)	0.63 (0.58, 0.68)	0.61 (0.56, 0.65)	0.65 (0.59, 0.69)

CN = cognitively normal; AD = Alzheimer's disease; MCI = mild cognitive impairment; CN* = cognitively normal at present but diagnosed with mild cognitive impairment in the future.

Dataset descriptions

- **ADNI**: Data used in the preparation of this article were obtained from the Alzheimer's Disease Neuroimaging Initiative (ADNI) database (adni.loni.usc.edu). The ADNI was launched in 2003 as a public-private partnership, led by Principal Investigator Michael W. Weiner, MD. The primary goal of ADNI has been to test whether serial magnetic resonance imaging (MRI), positron emission tomography (PET), other biological markers, and clinical and neuropsychological assessment can be combined to measure the progression of mild cognitive impairment (MCI) and early Alzheimer's disease (AD). Information about the dMRI sequence used in the present study is provided below. For further details, please visit https://adni.loni.usc.edu/

TE (s)	TR (s)	Number of directions (b=0)	Shells	Manufacturer	Model type	Field Strength
0.08	14.2	46 (5)	0, 1000	GE	Signa HDxt	3T
0.06	9.1	46 (5)	0, 1000	GE	Discovery MR750	3T
0.06	7.8	54 (6)	0, 1000	GE	Discovery MR750	3T
0.06	7.8	54 (6)	0, 1000	GE	Discovery MR750	3T
0.09	10.1	33 (1)	0, 1000	Philips	Achieva dStream	3T
0.06	9.0	36 (4)	0, 1000	GE	Signa Premier	3T
0.08	9.6	55 (7)	0, 1000	Siemens	Skyra	3T
0.06	7.2	55 (6)	0, 1000	Siemens	Prisma_fit	3T
0.06	7.8	54 (6)	0, 1000	GE	DISCOVERY MR750	3T
0.06	7.8	54 (6)	0, 1000	GE	DISCOVERY MR750	3T
0.08	15.3	36 (4)	0, 1000	GE	DISCOVERY MR750w	3T
0.07	13.0	46 (5)	0, 1000	GE	Signa HDxt	3T
0.07	3.4	127 (13)	0, 500, 1000, 2000	Siemens	Prisma	3T
0.06	7.2	55 (6)	0, 1000	Siemens	Prisma_fit	3T
0.07	3.4	127 (13)	0, 500, 1000, 2000	Siemens	Prisma	3T
0.10	10.9	36 (4)	0, 1000	Philips	Ingenia	3T
0.07	3.4	127 (12)	0, 500, 1000, 2000	Siemens	Prisma	3T
0.07	12.5	46 (5)	0, 1000	GE	Signa HDxt	3T
0.06	7.2	55 (7)	0, 1000	Siemens	Prisma_fit	3T
0.07	9.1	46 (5)	0, 1000	GE	DISCOVERY MR750	3T
0.06	9.1	46 (5)	0, 1000	GE	DISCOVERY MR750	3T

- **BIOCARD**: Information about the dMRI sequence used in the present study is provided below. For further details, please visit https://www.biocard-se.org/

TE (s)	TR (s)	Number of directions (b=0)	Shells	Manufacturer	Model type	Field Strength
0.075	7.5	33 (1)	0, 700	Phillips	Achieva	3T

- **BLSA**: Information about the dMRI sequence used in the present study is provided below. For further details, please visit https://blsa.nih.gov/

	Scanner A	Scanners B/C	Scanner D
Head coil	Philips 8-ch	Philips 8-ch	Philips 8-ch
Scan time (mins:secs)	3:56	3:58	4:20
Number of gradients	30	32	32
Number of b0 images	1	1	1
Max b-factor (s/mm²)	700	700	700
Number of signal averages (NSA)	1	1	1
Diffusion gradient timing DELTA/delta (ms)	39.2/15.1	36.3/16	36.3/13.5
Slice thickness (mm)	2.5	2.2	2.2
Number of slices	50	65	70
Flip angle (deg)	90	90	90
TR/TE (ms)	6210/80	6801/75	7454/75
Field of view (mm)	240x240	212x212	260x260
Acquisition matrix	96x96	96x95	116x115
Reconstruction matrix	256x256	256x256	320x320
Reconstructed voxel size (mm)	0.94x0.94	0.83x0.83	0.81x0.81

- **HCPA**: Data used in the preparation of this work were obtained from the Human Connectome Project (HCP) database (https://ida.loni.usc.edu/login.jsp). The HCP project (Principal Investigators: Bruce Rosen, M.D., Ph.D., Martinos Center at Massachusetts General Hospital; Arthur W. Toga, Ph.D., University of Southern California, Van J. Weeden, MD, Martinos Center at Massachusetts General Hospital) is supported by the National Institute of Dental and Craniofacial Research (NIDCR), the National Institute of Mental Health (NIMH) and the National Institute of Neurological Disorders and Stroke (NINDS). HCP is the result of efforts of co-investigators from the University of Southern California, Martinos Center for Biomedical Imaging at Massachusetts General Hospital (MGH), Washington University, and the University of Minnesota. (https://www.humanconnectome.org/study/hcp-lifespan-aging) Information about the imaging protocols can be found at https://www.humanconnectome.org/study/hcp-lifespan-aging/project-protocol/imaging-protocols-hcp-aging
- **ICBM**: Data used in the preparation of this work were obtained from the International Consortium for Brain Mapping (ICBM) database (www.loni.usc.edu/ICBM). The ICBM project (Principal Investigator John Mazziotta, M.D., University of California, Los Angeles) is supported by the National Institute of Biomedical Imaging and BioEngineering. ICBM is the result of efforts of co-investigators from UCLA, Montreal Neurologic Institute, University of Texas at San Antonio, and the Institute of Medicine, Juelich/Heinrich Heine University - Germany. (www.loni.usc.edu/ICBM)

- **NACC**: Information about the dMRI sequence used in the present study is provided below. For further details, please visit https://www.naccdata.org/

TE (s)	TR (s)	Number of directions (b=0)	Shells	Manufacturer	Model type	Field Strength
0.06	9.1	7 (1)	0, 1000	GE	DISCOVERY_MR750	3T
0.06	8.0	26 (1)	0, 1000	GE	DISCOVERY_MR750	3T
0.10	9.5	92 (80)	0, 1000	Siemens	TrioTim	3T
0.08	8.0	48 (8)	0, 1300	GE	DISCOVERY_MR750	3T
0.09	8.0	26 (2)	0, 1000	GE	GENESIS_SIGNA	1.5T
0.10	8.8	65 (1)	0, 3000	Phillips	Achieva	3T

- **OASIS3** and **OASIS4**: Information about the dMRI sequence used in the present study is provided below. For further details, please visit https://sites.wustl.edu/oasisbrains/

TE (s)	TR (s)	Number of directions (b=0)	Shells	Manufacturer	Model type	Field Strength
0.10	3.8	21 (3)	0, 1000	Siemens	Skyra	3T

- **ROSMAPMARS**: Information about the dMRI sequence used in the present study is provided below. For further details, please visit https://www.rushu.rush.edu/research/departmental-research/rush-alzheimers-disease-center/rush-alzheimers-disease-center-research/epidemologic-research

TE (s)	TR (s)	Number of directions (b=0)	Shells	Manufacturer	Model type	Field Strength
0.09	5.4	84 (12)	0, 900	GE	SIGNA_EXCITE	1.5T
0.09	8.1	46 (6)	0, 1000	Siemens	TrioTim	3T
0.09	8.1	46 (6)	0, 1000	Siemens	TrioTim	3T
0.09	8.1	46 (6)	0, 1000	Siemens	TrioTim	3T
0.05	12.0	41 (1)	0, 1000	PHILIPS-F398EB2	Achieva	3T
0.05	11.0	41 (1)	0, 1000	PHILIPS-IJI1EMU	Achieva_dStream	3T
0.05	11.5	41 (1)	0, 1000	PHILIPS-EKUR50U	Achieva_dStream	3T

- **UKBB**: The UK Biobank subset used in the present study includes all diffusion MRI and T1-weighted MRI scans approved and provided to us through application ID 16315 (Project title: Population Mapping of Brain, Eye, Spinal Cord, and Abdomen Anatomy in the Context of Electronic Medical Records), approved on April 6th, 2018. Information about the dMRI sequence used in the present study is provided below. For further details, please visit https://www.ukbiobank.ac.uk/

Resolution: 2x2x2 mm
Field-of-view: 104x104x72 matrix
Duration: 7 minutes (including 36 seconds phase-encoding reversed data)
5x b=0 (+3x b=0 blip-reversed), 50x b=1000 s/mm2, 50x b=2000 s/mm2
Gradient timings: δ=21.4 ms, Δ=45.5 ms; Spoiler b-value = 3.3 s/mm2 SE-EPI with x3 multislice acceleration, no iPAT, fat saturation

For the two diffusion-weighted shells, 50 distinct diffusion-encoding directions were acquired (and all 100 directions are distinct). The diffusion prepraration is a standard ("monopolar") Stejskal-Tanner pulse sequence. This enables higher SNR due to a shorter echo time (TE=92ms) than than a twice-refocused ("bipolar") sequence. This improvement comes at the expense of stronger eddy current distortions, which are removed in the image processing pipeline.

(https://biobank.ctsu.ox.ac.uk/crystal/crystal/docs/brain_mri.pdf)

- **VMAP**: Information about the dMRI sequence used in the present study is provided below. For further details, please visit https://www.vumc.org/vmac/vmap

TE (s)	TR (s)	Number of directions (b=0)	Shells	Manufacturer	Model type	Field Strength
0.06	10	37 (5)	0,1000	Philips	Achieva	3T

- **WRAP**: Information about the dMRI sequence used in the present study is provided below. For further details, please visit https://wrap.wisc.edu/

TE (s)	TR (s)	Number of directions (b=0)	Shells	Manufacturer	Model type	Field Strength
0.07	8.0	48 (8)	0, 1300	GE	DISCOVERY MR750	3T
